# Impact of Genetic Polymorphisms on the Smoking-related Risk of Periodontal Disease: the Population-based Study SHIP

**DOI:** 10.1186/1617-9625-1-3-197

**Published:** 2003-09-15

**Authors:** P Meisel, G Heins, LE Carlsson, J Giebel, U John, C Schwahn, T Kocher

**Affiliations:** 1 Department of Pharmacology, Ernst Moritz Arndt University Greifswald, Greifswald, Germany; 2 Department of Immunology, Ernst Moritz Arndt University Greifswald, Greifswald, Germany; 3 Department of Anatomy, Ernst Moritz Arndt University Greifswald, Greifswald, Germany; 4 Department of Epidemiology, Ernst Moritz Arndt University Greifswald, Greifswald, Germany; 5 Department of Periodontics, Ernst Moritz Arndt University Greifswald, Greifswald, Germany

## Abstract

Periodontitis is a bacterial inflammatory disease leading to attachment loss with the consequence of tooth loss. There exists a multifactorial risk pattern including bacterial challenge, smoking, age, sex, diabetes, socio-economic and genetic factors. Smoking has the highest impact on the course of the disease modulated by all the other factors. Here, we report the relationship between smoking and the polymorphisms of genetic polymorphisms inflicted in the pathogenesis.

In a randomly selected population-based study, 1083 subjects were typed for the polymorphisms of the IL-1 genotype, Fcγ RIIIb receptor gene, myeloperoxidase and N-acetyltransferase (NAT2) and related to their periodontal state. Smoking behavior was assessed including present and past quality and quantity of smoking.

There is a significant dose-effect relationship between the exposure to tobacco smoke and the extent of periodontal disease assessed as attachment loss and tooth loss. Moreover, there are gene-environmental interactions as subjects bearing variant genotypes show an enhanced smoking-associated risk of the disease modulated by these genotypes. In non-smokers, the impact of these genetic polymorphisms is mostly negligible.

This study provides support for the hypothesis that subjects bearing genetic variants of polymorphically expressed phenotypes are at an increased risk of periodontitis when smoking. Mostly, this may be accomplished via the influence of smoking-related impairment on defense mechanisms rather than on the pathogenic pathways.

## Introduction

Periodontitis is a very common inflammatory disease caused by oral bacteria and leading to irreversible attachment loss, bone destruction and eventually to tooth loss. Approximately 30% of the adults in Europe are affected, among them 5–15% with severe periodontal disease [1]. Similar figures were reported for the U.S.A. [2]. An interest in risk assessment for dental conditions came from the observation that some people are more likely to be affected by the sequelae of periodontitis than others [3]. Whereas the infection is a necessary prerequisite for the development of periodontitis, its course and severity depend on a number of inherited and environmental conditions. Thus, periodontal diseases present a wide range of clinical variability and severity. Both environmental and genetic factors contribute to individual variations in the etiology of periodontal diseases [4]. This individual susceptibility seems to be of major importance in determining the manifestation and progression of the disease [5]. Now there exists evidence that the inter-individual variability in this condition depends on genetic factors, probably most of them as yet unidentified [6,7].

Smoking is one of the major environmental risk factors of periodontitis as shown in numerous studies (for reviews see [8,9]). In different studies, smoking was confirmed as a risk factor for periodontitis with odds ratios varying between 2.5 and 6 [10]. In subgroups of patients the risk may be even higher, especially in the younger. Smoking is not only a risk factor for the severity of the disease, but smoking also delays healing and is associated with refractory periodontitis. Although the correlation between tobacco use and periodontal disease is quite strong, the role of tobacco in the pathogenesis of periodontal disease is uncertain. Environmental-gene interactions may play some role in the risk of the disease. Candidates for such genetic susceptibility factors are polymorphisms of genes modulating the immune response (e.g. FcγRIII receptors [11,12]), genes inflicted in metabolism of products of tobacco smoke (Myeloperoxidase [13], N-acetyltransferase [14]), genes in the process of inflammation (interleukin-1 [15,16]) or related to tissue destruction (metalloproteinases [17]).

**Figure 1:**
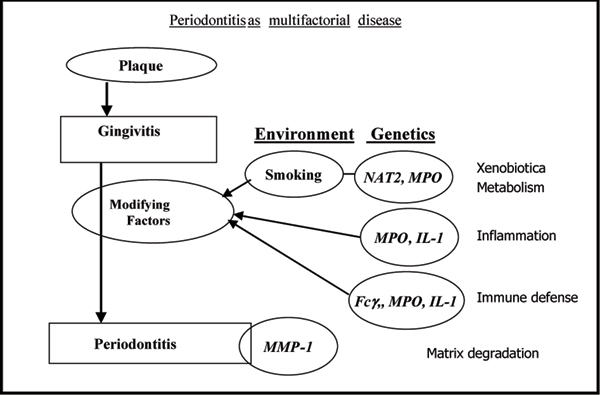
**Periodontitis as multifactorial disease: development of periodontal disease from bacterial challenge producing plaque to severe disease**. Abbreviations: Fcγ: Fcγ-receptors; IL-1: polymorphisms of interleukin-1 clusters; MMP-1: metalloproteinase-1 (collagenase); MPO: myeloperoxidase; NAT2: N-acetyltransferase 2.

All these genes are expressed polymorphically, i.e. at least two different types of the gene product exist in a population in a high and constant proportion. Consequently, the proteins expressed by the wildtype or mutated genes function differently in the pathogenesis of diseases influenced by them. A schematic representation of the course of periodontitis including modifying risk factors is depicted in Fig. 1. N-acetyltransferase (NAT2) and myeloperoxidase (MPO) are enzymes participating in the metabolism of xenobiotics including arylamines from tobacco smoke. In addition, MPO is inflicted in defense against bacterial challenge and inflammatory tissue destruction. Interleukin (IL-1) proteins play a pivotal role in chronic inflammation and function as stimulators of matrix destruction and bone resorption leading to tooth loss. Leukocyte Fcγ receptors mediate the effects of immunoglobulins and the variant genotypes express phenotypes of diminished affinity for IgG. They are expressed on neutrophils, macrophages, monocytes etc, all of them cell types invading inflamed tissues as the periodontal gingiva.

Numerous studies on the above mentioned and other genetic factors revealed risk-modulating effects for periodontal diseases. However, it is quite unclear if these effects alter the periodontal phenotype as such or if the action of other risk factors is modulated, affecting the periodontal outcome in an indirect way. As smoking is the most important risk factor for periodontitis, the objective of this study is to evaluate the interaction between smoking and various genetic factors known to have an impact on the course and severity of periodontitis. In a population-based, cross-sectional study we performed genotyping for different polymorphisms and related them to the smoking behavior of the participants.

## Materials and methods

### Subjects

3148 subjects were randomly selected from a population of 210,000 inhabitants of the German part of Pomerania in a study designated as SHIP (Study of Health In Pomerania). The design of the study, recruiting of participants and the scope of this population-based cross-sectional health survey were outlined elsewhere [18]. In this group, the most profound smoking-related differences in periodontal parameters were recognized between the ages of 40–60. Therefore, all subjects in the age range of 40 – 60 years (N = 1103) were included in the study reported here. Characteristics of these subjects, relevant to the objective of the study, are displayed in Table 1.

**Table 1:** Subjects enrolled in the Study of Health in Pomerania (SHIP) and assessed for periodontitis and their smoking state. Shown: the numbers of subjects, ratios, or the median and range of parameters where appropriate

	**Never smoked**	**Ever smoked**	**Current smokers**
Number of subjects	559	544	298
Female/male	388/171	206/338	164/134
Age, mean ± SD (range 40 – 60)	51.3 ± 5.7	49.7 ± 5.8	48.8 ± 5.7
Number of teeth, median *	22	21	19
% Attachment loss ≥ 3 mm, median	48.2	68.7	75.0
% Attachment loss ≥ 4 mm, median	18.8	34.0	47.5
% Attachment loss ≥ 6 mm, median	0	5.0	8.0
% Probing depth ≥ 4 mm	6.1	12.5	17.9
Probing depth, mean mm	2.5 ± 0.7	2.8 ± 0.8	2.9 ± 0.8
Diabetes	28 (5.0%)	38 (7.0%)	15 (5.0%)
High School = yes	101 (18.1%)	87 (16.0%)	40 (13.4%)

*except 3^rd ^molars

### Clinical status

The smoking status and habits of all subjects were assessed by an extensive questionnaire comprising 31 items concerning present and past quality and quantity of smoking. The items included questions as to whether the subjects had ever used tobacco products, the age at which they started smoking (and, if applicable, ceased smoking) and the frequency and duration of smoking. The dose smoked was registered as 'packyears', i.e. the packs of cigarettes smoked daily multiplied by the years of reported smoking. Another approach distinguished between subjects who never smoked and former smokers from those who are current smokers. In this study smokers were classified as regularly smokers when smoking at least 1 cigarette/day. The group of subjects designated as 'ever smokers' comprised current and former (quitted) smokers. In an independent study (n = 335), we proved by cotinine determinations that no more than 2–3% of the subjects gave answers not consistent with their true smoking behavior. Investigations in representative population samples provided evidence that self-reported smoking status is accurate [19]. The periodontal state was assessed by trained dentists. Assessment included probing depth, attachment loss, bleeding and plaque index. The periodontal examination was carried out on either the left or right quadrants and the examination side was changed from subject to subject. All fully erupted teeth were assessed excluding third molars. A maximum of 14 teeth per subject was examined. Attachment loss and probing depth were assessed with a periodontal probe (PCP 11, HuFriedy, Chicago IL.) at mesiobuccal, distobuccal, midbuccal and midlingual aspect on each selected tooth. The measurements were made in whole millimeters.

### Genotyping

Genomic DNA was extracted from leukocytes isolated from venous blood. Of the 1103 subjects enrolled, 1085 were successfully genotyped for the variants of the IL-1 gene cluster. The remaining refused DNA analyses, or genotyping failed. Genotyping was performed at positions IL-1B +3954, IL-1B -511 and IL-1A -889 according to methods based on PCR and restriction enzyme digestion as described elsewhere [20]. Sequences of the primers employed were the same as described for IL-1A -889 in [21], IL-1B -511 and IL-1B +3954 in [20]. Restriction enzymes used were purchased from BioLabs (Schwalbach, Germany): Nco I (IL-1A -889), Ava I (IL-1B -511), Taq I (IL-1B +3954). By general agreement, subjects with at least one variant allele of each IL-1A and IL-1B were designated as 'genotype positive'. This is the composite genotype suggested by Kornman et al. [20] to be a susceptibility factor for an enhanced periodontitis risk.

Of the 1103 subjects enrolled, 1083 were successfully genotyped for MPO polymorphism. Genotyping for MPO G-463A was performed by PCR/RFLP according to the method described by Cascorbi et al. [22]. For PCR we used the primers forward 5'-CGG TAT AGG CAC ACA ATG GTG AG, reverse 5'-GCA ATG GTT CAA GCG ATT CTT C; the amplified PCR fragment was digested with endonuclease AciI in order to distinguish the genotypes wild-type -463 G/G, heterozygous G/A, and homozygous A/A.

The coding region DNA sequence of the NAT2 gene was amplified by PCR, essentially as previously described [13]. Mutations at positions T341C, G590A, and G857A were detected by restriction fragment length differences (RFLP) or allele specific PCR as described. In order to predict the phenotype from genotyping, subjects bearing one or both alleles NAT2*4 (dominant wild-type) were considered as 'rapid' acetylators, those with two variant alleles as 'slow' acetylators [24]. As most NAT2 variants involve two or three point mutations, 'homozygous' refers to allele combinations rather than point mutations.

The polymorphic sites of the FcγRIIIa, and – RIIIb genes were determined also by PCR methodology. The methods for genotyping were essentially as described; FcγRIIIa-158 V/F [25] and FcγRIIIb-NA1/NA2 [26], respectively.

### Statistics

**Figure 2:**
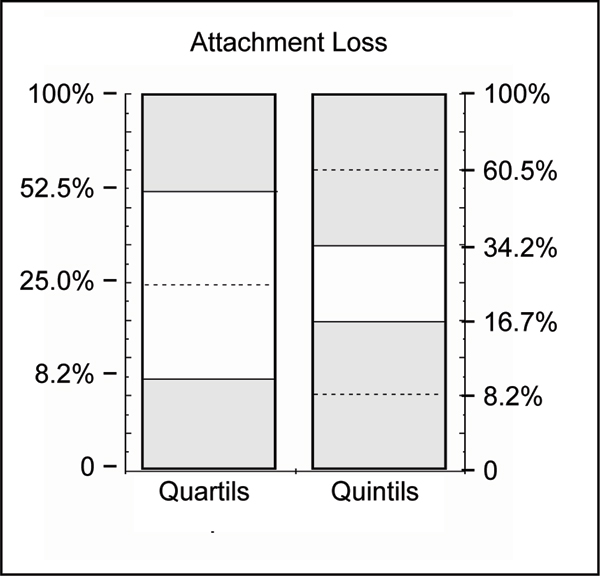
**Illustration of separation criteria between periodontally 'diseased' and 'healthy' subjects, indicated by the shaded areas**. Quantils to dichotomize the continuous variable % attachment loss into 'cases' (upper quantils) versus 'controls' lower quantils. Numbers on the ordinate give the cut-offs as attachment loss ≥ 4 mm in percent of the sites measured.

Periodontal disease occurs as a continuous range from minor signs to severe disease, complicated by the variability between different subjects and between the teeth within a particular individual. Thus, in order to avoid any arbitrarily chosen disease criteria or cut-offs, we used strictly statistical methods to distinguish periodontally 'healthy' from 'diseased' subjects. To differentiate the extent of attachment loss (percent of sites exceeding 4 mm) quantils (quartils or quintils) were calculated and subjects in the upper quantils of distribution ('cases') were compared with those in the lower ones with no or minor attachment loss ('controls'). This method is explained in Fig. 2. Most severe cases of periodontitis were defined as subjects with an extent of attachment loss ≥ 6 mm in the uppermost decentil (more than 40%).

Logistic regression analyses were performed by STATVIEW^® ^(SAS Institute, Cary, NC). ANOVA and contingency table distributions were also used as well as the Kruskal-Wallis test for the comparison of differences occurring between three genotypes.

## Results

**Figure 3:**
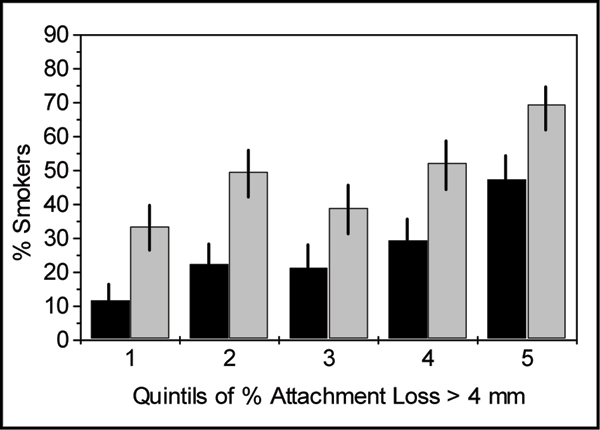
**Proportion of smokers among subjects of increasing extent of periodontal disease as shown by quintiles according to Fig. 2**. Black bars: current smokers only, gray bars: current and quitted smokers (ever).

As shown in Table 1, smokers have more severe periodontitis and a higher extent of the disease compared to subjects who never smoked. This is not attributable to age, sex, education or diabetes – all other risk factors of periodontal diseases. Doubtless, the higher the extent of periodontal disease the higher the number of smokers is in the respective group of subjects. The ratio of smokers to non-smokers is steadily increasing from the group with no or slight signs of the disease (first quintil in Fig. 3) to that with a high extent of attachment loss (5th quintil). The smoking-associated risk for the upper quintil of measured attachment loss increased sevenfold as compared to the lowest quintil, i.e. the crude odds ratios were 6.88 (95% confidence interval 4.15 – 11.41, p < 0.0001) and 4.51 (95% C.I. 2.98 – 6.82, p < 0.0001) for current and ever (i.e. present and quitted) smokers, respectively.

In the Caucasian population studied here, the assessed gene frequencies including the 95% confidence intervals were for NAT2 – slow acetylating phenotype 57.1% (54.1 – 60.0), MPO low expression A/A – A/G genotypes 34.1% (31.1 – 36.9), FcγRIIIa-158F low IgG affinity 57.4% (54.4 – 60.3), FcγRIIIb-NA2 – low affinity 86.9% (84.8 – 88.8) and IL-1 – risk (positive) genotype 36.4% (33.6 – 39.3). These frequencies are in agreement with other studies made in Caucasian populations.

From an epidemiological point of view, considering the frequencies of smoking and the putative genetic factors as well as the prevalence of periodontitis, an interaction between these factors seems reasonable. In the following, it will be shown that each of these genetic polymorphisms has an impact on the extent of periodontal disease and that it is mediated by the interaction with smoking.

**Figure 4:**
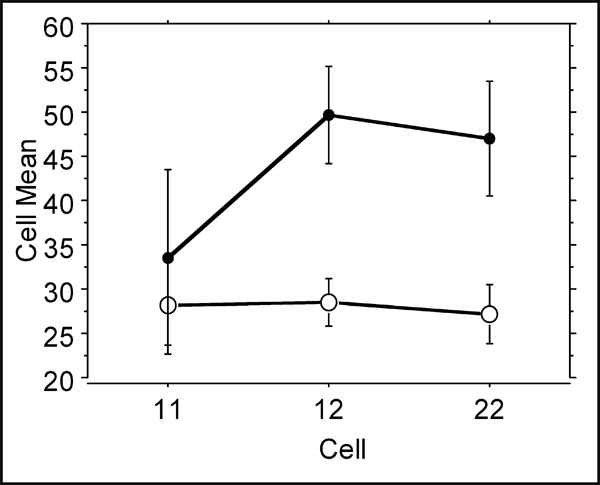
**Percent attachment loss ≥ 4 mm among smokers (filled circles) and non-smokers (open circles) separated according to their Fcγ-RIIIb genotypes NA1/NA1, NA1/NA2 or NA/2NA2**. Kruskal-Wallis test results in p = 0.024 and p = 0.797 for smokers and non-smokers, respectively.

Leukocyte Fcγ receptors mediate the effects of immunoglobulins induced for defense against periodontopathogenic bacteria. Whereas in this study no effect was seen with the different FcγRIIIa types, the FcγRIIIb influence the disease parameters. In Fig. 4, the different genotypes show no differences in non-smokers. However, smokers show a significantly increased attachment loss when heterozygous or homozygous for the variant low affinity allele FcγRIIIb-NA2.

**Figure 5:**
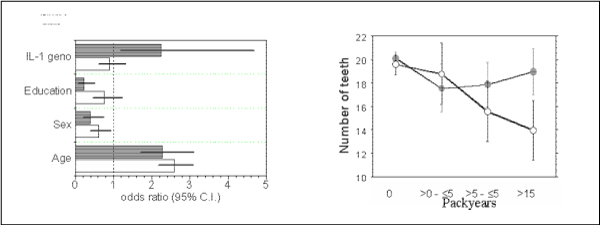
**Impact of IL-1 genotype on risk of periodontitis**. ***5a (left)***: Results of logistic regression analysis for non-smokers (open bars) and smokers (shaded bars). Shown are the odds ratios including the 95% confidence intervals (C.I.). ***5b (right)***: Mean number of teeth in subjects of different smoking exposure as given at the abscissa. Open symbols: IL-1 genotype positive subjects, closed: IL-1 genotype negative. ANOVA packyears p < 0.0001, genotype p = 0.015, interaction packyears*genotype p = 0.013.

Likewise, subjects bearing the 'risky' genotype of interleukin-1 (at least one variant allele of each IL-1A and IL-1B) belong to the group with an enhanced attachment loss only when smoking. Non-smokers do not differ in their periodontal parameters irrespective of the IL-1 genotype. This is illustrated by the results of logistic regression analyses as shown in Fig. 5a. Attachment loss is dependent on the age of the test persons. The probability of disease increases considerably for smokers and, beyond this, in smokers who are genotype-positive for the IL-1 polymorphism. Moreover, an exposure-effect relation can be demonstrated in this gene-environmental interaction. Attachment loss is an indicative symptom of periodontal disease, but tooth loss is the final result. Even though tooth loss is a highly variable parameter due to different fates of particular teeth (caries, dentists decisions, accidents), the gene-dose effect is shown with the number of teeth remaining (Fig. 5b). Again, an increasing exposure to tobacco smoke is associated with increasing effects indicated by the diminished number of remaining teeth. The tooth loss is reinforced in subjects bearing the IL-1 positive genotype. There are no differences in the mean ages, so the higher figures of pack-years are not biased by higher age.

Polymorphic variants of both N-acetyltransferase (NAT2) and myeloperoxidase (MPO) affect the extent of the periodontal state, especially attachment loss. Subjects designated as so-called 'rapid acetylators' (bearing at least one wild-type allele NAT2*4) or bearing the highly expressed MPO variant (homozygous -463 G/G) are at an increased risk of periodontitis when smoking. However, as non-smokers the rapid acetylating NAT2 genotypes are at a lower risk. This is in contrast to MPO with a lower risk in subjects bearing the low expression variants A/G or A/A. This is shown in Table 2 by the frequency distribution of the most severe periodontitis cases exhibiting more than 6 mm attachment loss.

**Table 2:** Impact of gene polymorphisms of enzymes participating in the metabolism of tobacco smoke products: N-acteyltransferase 2 (NAT2) and myeloperoxidase (MPO). Shown is the distribution of subjects with severe periodontitis (more than 30% of sites have attachment loss > 6 mm) versus those having no attachment loss ≥ 6 mm. NAT2 high activity: homo- or heterozygous with allele NAT2*4; MPO highly expressed variant coded by homozygous G/G subjects. Shown are crude odds ratios (95% confidence intervals, C.I.)

		***NAT2***			***MPO***		
**Smoking**	**High activity variant**	**AV6 mm > 30%**	**AV6 mm = 0**	**odds ratio**	**AV6 mm > 30%>**	**AV6 mm = 0**	**odds ratio**

never	present	22	132	1.0 (ref.)	38	160	1.0 (ref.)
never	no	29	128	1.4 (0.7 – 2.6)	13	100	0.5 (0.3 – 1.1)
current	no	38	54	4.2 (2.2 – 8.2)	21	67	2.9 (1.5 – 6.0)
current	present	28	24	7.0 (3.3 – 15.2)	45	114	4.0 (2.2 – 7.0)

## Discussion

To date, the complex interaction of environmental factors with periodontal diseases is poorly understood. Although the correlation between tobacco use and periodontal disease is quite strong, the role of tobacco in the pathways of periodontal disease is uncertain [27]. In addition, there is obviously an interaction between hereditary factors and smoking, the latter being also associated with sex, age, education, stress and other risk factors of periodontitis.

With respect to smoking, direct local effects are to be distinguished from systemic effects. Complex interactions between circulatory and immunological effects exerted by nicotine as well as toxic effects by arylamines and other products of tobacco smoke are to be taken into consideration. Thus, it seems plausible to study genetic dispositions of inflammation-related factors as well as toxicity-modulating metabolic polymorphisms.

As can be concluded from the results presented, smoking is the most important risk factor: it increases the risk of periodontitis irrespective of the genotype. This risk is further aggravated in subjects bearing particular alleles of the polymorpically expressed genes studied. The risk of the disease may be worsened by either the genetic or the environmental agent, possibly the genotype increases the effect of the agent [28]. Smoking periodontitis subjects typically failed to respond to specific microbes [29]. Arylamines when detoxified insufficiently act as immunosuppressants [30], they are activated to reactive intermediates in leukocytes or by activated neutrophils and monocytes.

The binding of IgG immune complexes by phagocytes is an important defense mechanism of inflammation. The activation of the FcγRIII receptors mediates the release of cytokines, phagocytosis and cell-mediated cytotoxicity [31]. The polymorphism of FcγRIIIb influences the ability of neutrophils to phagocytize IgG-opsonized particles. The finding of an association between the frequency of the FcγRIIIb allele of low IgG affinity and the extent of periodontitis is in agreement with studies by Kobayashi et al. [12].

This receptor isoform exhibits a lower affinity toward IgG1 and IgG3. As shown (Fig. 4), smoking and the presence of the FcγRIIIb NA2 allele leads to an increased attachment loss indicating more severe disease.

This is in agreement with results showing that smoking lowered the sensitivity to the stimulation of Fcγ receptors [32]. Ligation of FcγR causes the release of inflammatory mediators, among them interleukin IL-1.

Phenotypic differences exist in interleukin levels corresponding to the genotype and these differences are related to periodontal disease [33]. The positive IL-1 genotype results in enhanced levels of pro-inflammatory cytokines and an increased formation of interleukins is known to be induced by nicotine and/or bacterial lipopolysaccharides [34,35]. These changes may serve as predisposing factors in periodontal disease. IL-1 gene polymorphisms are highly related to plasma levels of CRP and fibrinogen which are markers of systemic inflammation [36]. Frequently, such signs of systemic inflammation are enhanced in smokers. Different studies revealed the suppression of cytokine production by tobacco smoke extracts [37]. Release of cytokines from neutrophils seems to be affected more by smoking than the disease.

However, in gingival crevice fluid no difference was found in IL-1β concentrations [38].

It is well known that myeloperoxidase activity is increased in inflammatory gingival tissue. Inflammation-related recruitment of neutrophil leukocytes into the affected tissue is most likely responsible for the action of MPO in the gingiva. Exchange of an adenosine for a guanosine at position – 463 in the 5'-untranslated region of the MPO gene leads to the loss of a transcription factor binding site. Reduced binding of transcription factor SP1 results in a diminished expression of MPO. Smoking may override this protection of diminished enzyme activity in the most affected individuals (more than 30% attachment loss 6 mm, Table 2). However, in subjects with more moderate disease, the protective effect is still detectable in smokers also [13]. Possible protective effects seen in non-smokers bearing at least one A allele of MPO are not obvious in smokers. Probably, the MPO polymorphism-related effects are sex-specific as the protective role of the -463A allele was detected in women but not in male subjects [13].

Smoking subjects who were phenotypically rapid acetylators had more severe signs of periodontal disease than non-smoking patients regardless if phenotypically rapid or phenotypically slow. As shown, only subjects bearing both risk factors – smoking and metabolic rapid phenotype – are prone to increased attachment loss. These data suggest that the increase in risk imposed by NAT2 acetylation may be related to smoking. Again, this influence was obvious only in the most affected individuals. In a former study, we have shown that, in contrast, smoking slow acetylators bear a higher risk when bone loss was the parameter assessed to characterize the disease [14]. Any explanation of the association between the acetylation polymorphism and the immune/inflammatory responses remains speculative at this time. The known role of the enzyme NAT2 is related to the detoxification of foreign substances in the liver. Nicotine induces a local vasoconstriction reducing blood flow and gingival bleeding. On a systemic level, smoking leads to diminished levels of circulating IgG2, the immunoglobulin reactive with periodontitis-associated bacteria [29]. Systemic alterations in the host response by genetic variations in the N-acetyltransferase metabolic capacity for arylamines may explain the mechanism for the results shown here. Possibly, the impaired metabolism of smoke-derived arylamines may have relations to other known risk factors, for instance the IgG2-related immune reactions. Arylamines may act as immunosuppressants [30]. They are activated to reactive intermediates in leukocytes by NAT [39], by activated neutrophils and monocytes releasing myeloperoxidase or even by tissue-specific activation by prostaglandin H synthase [40].

In conclusion, neutrophils are the most probable effector cells of the smoking-related effects associated with the genetic polymorphism shown. Recruitment of neutrophils and their exaggerated responses are a critical component in the pathogenesis of periodontal disease including local tissue injury [41]. Their function is impaired in smokers with chronic periodontitis [42]. The polymorphisms related to this central role represent major steps in this interplay as cell recruitment by IL-1, cell activation by IgG binding at Fcγ receptors or release of myeloperoxidase from the cellular granules. Impairment of these functions by different phenotypes seems to have only minor effects on the disease. The additional harmful tobacco smoke may override the potential for compensation. The extent of such interplay may depend on quantitative dose-effect relationships. A recent study suggests dose-effect relationships exist with respect to the genetic as well as the environmental factor [43].

## Competing interests

The authors declare that they have no competing interests.
